# 1,2-Diazinium hydrogen chloranilate

**DOI:** 10.1107/S1600536808031978

**Published:** 2008-10-11

**Authors:** Kazuma Gotoh, Hiroyuki Ishida

**Affiliations:** a Department of Chemistry, Faculty of Science, Okayama University, Okayama 700-8530, Japan

## Abstract

In the crystal structure of the title compound, C_4_H_5_N_2_^+^·C_6_HCl_2_O_4_^−^, there are three crystallographically independent 1,2-diazinium cations and hydrogen chloranilate anions. The anions are held together by pairs of O—H⋯O hydrogen bonds to form two types of dimers, one of which is centrosymmetric. The 1,2-diazinium cations are linked on both sides of each dimer *via* bifurcated N—H⋯O hydrogen bonds to give two kinds of 2–2 cation–anion associations. The 2–2 associations are linked by inter­molecular C—H⋯O and C—H⋯N hydrogen bonds, forming a mol­ecular tape along the [230] direction. The tapes are further connected by C—H⋯O hydrogen bonds, forming a three-dimensional network.

## Related literature

For general background, see: Gotoh *et al.* (2007 ▶). For related compounds, see: Gotoh *et al.* (2008 ▶); Ishida & Kashino (1999 ▶).
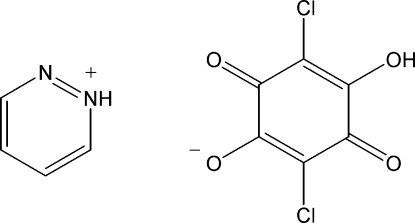


## Experimental

### 

#### Crystal data

C_4_H_5_N_2_^+^·C_6_HCl_2_O_4_^−^
*M* *_r_* = 289.07
Monoclinic, 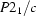

*a* = 25.6849 (10) Å
*b* = 7.0516 (2) Å
*c* = 18.1388 (6) Å
β = 97.5822 (13)°
*V* = 3256.56 (18) Å^3^
*Z* = 12
Mo *K*α radiation
μ = 0.61 mm^−1^
*T* = 173 (1) K
0.40 × 0.22 × 0.12 mm

#### Data collection

Rigaku R-AXIS RAPIDII diffractometer
Absorption correction: numerical (*ABSCOR*; Higashi, 1999 ▶) *T*_min_ = 0.855, *T*_max_ = 0.930
42942 measured reflections
9398 independent reflections
7981 reflections with *I* > 2σ(*I*)
*R*_int_ = 0.025

#### Refinement

*R*[*F* ^2^ > 2σ(*F* ^2^)] = 0.030
*wR*(*F* ^2^) = 0.082
*S* = 1.04
9398 reflections
511 parameters
H atoms treated by a mixture of independent and constrained refinement
Δρ_max_ = 0.47 e Å^−3^
Δρ_min_ = −0.26 e Å^−3^

### 

Data collection: *PROCESS-AUTO* (Rigaku/MSC, 2004 ▶); cell refinement: *PROCESS-AUTO*; data reduction: *CrystalStructure* (Rigaku/MSC, 2004 ▶); program(s) used to solve structure: *SHELXS97* (Sheldrick, 2008 ▶); program(s) used to refine structure: *SHELXL97* (Sheldrick, 2008 ▶); molecular graphics: *ORTEP-3* (Farrugia, 1997 ▶); software used to prepare material for publication: *CrystalStructure* and *PLATON* (Spek, 2003 ▶).

## Supplementary Material

Crystal structure: contains datablocks global, I. DOI: 10.1107/S1600536808031978/fj2158sup1.cif

Structure factors: contains datablocks I. DOI: 10.1107/S1600536808031978/fj2158Isup2.hkl

Additional supplementary materials:  crystallographic information; 3D view; checkCIF report

## Figures and Tables

**Table 1:** Hydrogen-bond geometry (Å, °)

*D*—H⋯*A*	*D*—H	H⋯*A*	*D*⋯*A*	*D*—H⋯*A*
N1—H1⋯O2	0.889 (17)	1.753 (17)	2.6138 (13)	162.2 (17)
N1—H1⋯O3	0.889 (17)	2.578 (18)	3.1583 (14)	123.7 (14)
N3—H3⋯O6	0.886 (18)	1.811 (17)	2.6621 (13)	160.4 (17)
N3—H3⋯O7	0.886 (18)	2.431 (18)	3.0066 (14)	123.0 (14)
O4—H4⋯O1	0.79 (2)	2.26 (2)	2.6619 (13)	113 (2)
O4—H4⋯O5	0.79 (2)	1.94 (2)	2.6723 (12)	155 (2)
N5—H5⋯O10	0.890 (18)	1.881 (18)	2.7450 (14)	163.2 (16)
N5—H5⋯O11	0.890 (18)	2.410 (17)	2.9659 (13)	120.7 (14)
O8—H8⋯O1	0.80 (2)	1.86 (2)	2.5930 (12)	152 (2)
O8—H8⋯O5	0.80 (2)	2.25 (2)	2.6582 (13)	112.7 (18)
O12—H12⋯O9	0.79 (2)	2.23 (2)	2.6595 (13)	115.1 (18)
O12—H12⋯O9^i^	0.79 (2)	2.02 (2)	2.6801 (12)	142 (2)
C20—H20⋯O2^ii^	0.95	2.57	3.1097 (15)	116
C21—H21⋯O3^iii^	0.95	2.36	3.2754 (15)	162
C22—H22⋯O3	0.95	2.60	3.1913 (15)	121
C25—H25⋯O10	0.95	2.47	3.4056 (15)	169
C26—H26⋯O7	0.95	2.44	3.0363 (15)	121
C26—H26⋯N6	0.95	2.51	3.2665 (17)	137
C27—H27⋯O7	0.95	2.37	3.2765 (15)	159
C28—H28⋯O6^iv^	0.95	2.47	3.1091 (15)	124
C30—H30⋯O11	0.95	2.34	2.9551 (15)	122

Symmetry codes: (i) 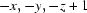; (ii) 
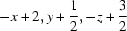
; (iii) 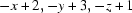; (iv) 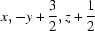.

**Fig. 1.**
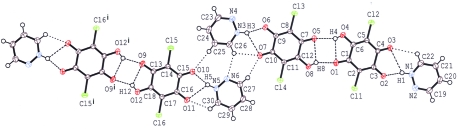
The structure of the 2:2 units of (I). The dashed lines indicate O—H···O, C—H···O and C—H···N hydrogen bonds (symmetry code as Table 1). Displacement ellipsoids of non-H atoms are drawn at the 50% probability level.

**Fig. 2.**
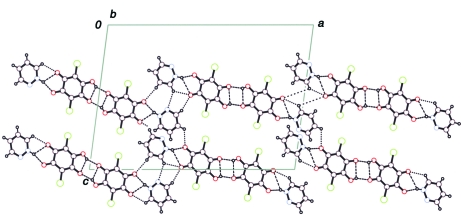
A partial packing diagram of (I), viewed down the b axis. The dashed lines indicate O—H···O, C—H···O and C—H···N hydrogen bonds.

## supplementary crystallographic information

### Comment

The title compound, (I), was prepared in order to extend our study on *D*—H···*A* hydrogen bonding (*D* = N, O, or C; *A* = N, O or Cl) in amine–chloranilic acid (systematic name: 2,5-dichloro-3,6-dihydroxy-1,4-benzoquinone) systems (Gotoh *et al.*, 2007). The crystal structure of 1,2-diazine (pyridazine)–chloranilic acid (2/1) was already reported (Ishida & Kashino, 1999; Gotoh *et al.*, 2008).

The asymmetric unit in (I) contains three 1,2-diazinium cations and three hydrogen chloranilate anions. Hydrogen chloranilate anions are held together by O—H···O hydrogen bonds (Table 1) to form two crystallographically independent dimers. The 1,2-diazinium cations are linked on both sides of each dimer *via* bifurcated N—H···O hydrogen bonds to give centro- and acentrosymmetric 2:2 salts of 1,2-diazine and chloranilic acid (Fig. 1). The N···O distances [2.6138 (13)–3.1583 (14) Å] in the hydrogen bonds are longer than those [2.582 (3) Å at 299 K (Ishida & Kashino, 1999) and 2.5549 (12) Å at 110 K (Gotoh *et al.*, 2008)] in 1,2-diazine–chloranilic acid (2/1) and the H atoms are located at the N atom sites. The dihedral angles between C1–C6 and C7–C12 rings, C1–C6 and N1/N2/C19–C22 rings, C7–C12 and N3/N4/C23–C26 rings, and C13–C18 and N5/N6/C27–C30 rings are 3.40 (5), 11.83 (5), 4.88 (6) and 11.41 (5)°, respectively. The 2:2 units are linked by intermolecular C—H···O and C—H···N hydrogen bonds, forming a molecular tape along the [230] direction. The tapes are further connected by C—H···O hydrogen bonds, forming a three-dimensional network (Table 1 and Fig. 2).

### Experimental

Single crystals were obtained by slow evaporation from a methanol solution (30 ml) of chloranilic acid (400 mg) and 1,2-diazine (155 mg).

### Refinement

C-bound H atoms were positioned geometrically (C—H = 0.95 Å) and refined as riding, with *U*_iso_(H) = 1.2*U*_eq_(C). H atoms in the O—H···O and N—H···O hydrogen bonds were located in a difference Fourier map and refined isotropically (refined distances given in Table 1).

### Crystal data

**Table:**

C_4_H_5_N_2_^+^·C_6_HCl_2_O_4_^−^	*F*(000) = 1752.00
*M**_r_* = 289.07	*D*_x_ = 1.769 Mg m^−^^3^
Monoclinic, *P*2_1_/*c*	Mo *K*α radiation, λ = 0.71075 Å
Hall symbol: -P 2ybc	Cell parameters from 36292 reflections
*a* = 25.6849 (10) Å	θ = 3.0–30.0°
*b* = 7.0516 (2) Å	µ = 0.61 mm^−^^1^
*c* = 18.1388 (6) Å	*T* = 173 K
β = 97.5822 (13)°	Prism, brown
*V* = 3256.56 (18) Å^3^	0.40 × 0.22 × 0.12 mm
*Z* = 12	

### Data collection

**Table:**

Rigaku R-AXIS RAPIDII diffractometer	7981 reflections with *I* > 2σ(*I*)
Detector resolution: 10.00 pixels mm^-1^	*R*_int_ = 0.025
ω scans	θ_max_ = 30.0°
Absorption correction: numerical (ABSCOR; Higashi, 1999)	*h* = −36→36
*T*_min_ = 0.855, *T*_max_ = 0.930	*k* = −9→8
42942 measured reflections	*l* = −24→25
9398 independent reflections	

### Refinement

**Table:**

Refinement on *F*^2^	Primary atom site location: structure-invariant direct methods
Least-squares matrix: full	Secondary atom site location: difference Fourier map
*R*[*F*^2^ > 2σ(*F*^2^)] = 0.030	Hydrogen site location: inferred from neighbouring sites
*wR*(*F*^2^) = 0.082	H atoms treated by a mixture of independent and constrained refinement
*S* = 1.04	*w* = 1/[σ^2^(*F*_o_^2^) + (0.0465*P*)^2^ + 0.8542*P*] where *P* = (*F*_o_^2^ + 2*F*_c_^2^)/3
9398 reflections	(Δ/σ)_max_ = 0.002
511 parameters	Δρ_max_ = 0.47 e Å^−^^3^
0 restraints	Δρ_min_ = −0.26 e Å^−^^3^

### Special details

**Table:**

Geometry. All e.s.d.'s (except the e.s.d. in the dihedral angle between two l.s. planes) are estimated using the full covariance matrix. The cell e.s.d.'s are taken into account individually in the estimation of e.s.d.'s in distances, angles and torsion angles; correlations between e.s.d.'s in cell parameters are only used when they are defined by crystal symmetry. An approximate (isotropic) treatment of cell e.s.d.'s is used for estimating e.s.d.'s involving l.s. planes.
Refinement. Refinement of *F*^2^ against ALL reflections. The weighted *R*-factor *wR* and goodness of fit *S* are based on *F*^2^, conventional *R*-factors *R* are based on *F*, with *F* set to zero for negative *F*^2^. The threshold expression of *F*^2^ > σ(*F*^2^) is used only for calculating *R*-factors(gt) *etc*. and is not relevant to the choice of reflections for refinement. *R*-factors based on *F*^2^ are statistically about twice as large as those based on *F*, and *R*- factors based on ALL data will be even larger.

### Fractional atomic coordinates and isotropic or equivalent isotropic displacement parameters (Å^2^)

**Table:**

	*x*	*y*	*z*	*U*_iso_*/*U*_eq_	
Cl1	0.788813 (11)	1.00777 (4)	0.695195 (15)	0.02287 (7)	
Cl2	0.813873 (11)	1.33053 (4)	0.375637 (15)	0.01939 (6)	
Cl3	0.546309 (11)	0.98926 (4)	0.291828 (15)	0.02164 (7)	
Cl4	0.529070 (10)	0.68298 (4)	0.616552 (15)	0.01938 (6)	
Cl5	0.134170 (11)	0.31600 (4)	0.406398 (15)	0.02014 (6)	
Cl6	0.126530 (11)	0.00278 (4)	0.732528 (15)	0.02182 (7)	
O1	0.70886 (3)	1.03911 (14)	0.56092 (5)	0.02672 (19)	
O2	0.88525 (3)	1.17619 (12)	0.64597 (5)	0.02155 (17)	
O3	0.89647 (3)	1.29114 (12)	0.50763 (5)	0.02036 (16)	
O4	0.71996 (3)	1.15850 (13)	0.42465 (5)	0.02316 (18)	
O5	0.62655 (3)	0.99280 (14)	0.42628 (5)	0.0258 (2)	
O6	0.45198 (3)	0.80783 (12)	0.34672 (5)	0.02140 (17)	
O7	0.44599 (3)	0.69004 (12)	0.48544 (5)	0.02138 (17)	
O8	0.61970 (3)	0.86803 (14)	0.56295 (5)	0.02294 (18)	
O9	0.04325 (3)	0.14786 (13)	0.47288 (5)	0.02485 (19)	
O10	0.22343 (3)	0.29297 (12)	0.53149 (5)	0.02021 (17)	
O11	0.21688 (3)	0.17839 (12)	0.67058 (5)	0.02290 (18)	
O12	0.04124 (3)	0.00464 (13)	0.60811 (5)	0.02206 (18)	
N1	0.97488 (4)	1.35579 (15)	0.65484 (6)	0.0219 (2)	
N2	0.99547 (4)	1.31774 (15)	0.72462 (6)	0.0247 (2)	
N3	0.36001 (4)	0.64298 (15)	0.35724 (6)	0.0212 (2)	
N4	0.33565 (4)	0.66986 (16)	0.28834 (6)	0.0265 (2)	
N5	0.31069 (4)	0.37842 (15)	0.63023 (6)	0.02006 (19)	
N6	0.34283 (4)	0.45730 (16)	0.58711 (6)	0.0239 (2)	
C1	0.75362 (4)	1.09400 (16)	0.55266 (6)	0.0170 (2)	
C2	0.79706 (4)	1.09715 (16)	0.60871 (6)	0.01556 (19)	
C3	0.84590 (4)	1.16825 (15)	0.59736 (6)	0.0152 (2)	
C4	0.85282 (4)	1.24116 (15)	0.51915 (6)	0.01446 (19)	
C5	0.80717 (4)	1.24251 (15)	0.46242 (6)	0.01550 (19)	
C6	0.76101 (4)	1.16835 (15)	0.47678 (6)	0.0164 (2)	
C7	0.58355 (4)	0.92184 (16)	0.43505 (6)	0.0165 (2)	
C8	0.54005 (4)	0.90299 (16)	0.37928 (6)	0.0157 (2)	
C9	0.49245 (4)	0.82418 (15)	0.39314 (6)	0.0155 (2)	
C10	0.48814 (4)	0.75267 (15)	0.47232 (6)	0.01508 (19)	
C11	0.53375 (4)	0.76601 (15)	0.52856 (6)	0.01517 (19)	
C12	0.57836 (4)	0.84830 (16)	0.51193 (6)	0.0162 (2)	
C13	0.08582 (4)	0.15642 (15)	0.51475 (6)	0.0164 (2)	
C14	0.13318 (4)	0.23100 (15)	0.49570 (6)	0.01558 (19)	
C15	0.17974 (4)	0.23689 (15)	0.54582 (6)	0.01537 (19)	
C16	0.17708 (4)	0.16726 (15)	0.62576 (6)	0.0154 (2)	
C17	0.12835 (4)	0.08899 (16)	0.64439 (6)	0.0159 (2)	
C18	0.08570 (4)	0.07922 (15)	0.59219 (6)	0.0156 (2)	
C19	1.04186 (5)	1.39362 (18)	0.74659 (7)	0.0234 (2)	
H19	1.0576	1.3710	0.7962	0.028*	
C20	1.06920 (5)	1.50533 (17)	0.70098 (7)	0.0232 (2)	
H20	1.1027	1.5564	0.7190	0.028*	
C21	1.04646 (5)	1.53937 (18)	0.62952 (7)	0.0231 (2)	
H21	1.0637	1.6133	0.5962	0.028*	
C22	0.99713 (5)	1.46098 (17)	0.60787 (7)	0.0228 (2)	
H22	0.9795	1.4834	0.5592	0.027*	
C23	0.28768 (5)	0.59892 (19)	0.27484 (7)	0.0279 (3)	
H23	0.2688	0.6165	0.2266	0.034*	
C24	0.26300 (5)	0.49954 (18)	0.32732 (8)	0.0250 (3)	
H24	0.2286	0.4497	0.3148	0.030*	
C25	0.28964 (5)	0.47601 (18)	0.39706 (7)	0.0252 (2)	
H25	0.2746	0.4101	0.4347	0.030*	
C26	0.33997 (5)	0.55323 (18)	0.41044 (7)	0.0247 (2)	
H26	0.3599	0.5409	0.4582	0.030*	
C27	0.38814 (5)	0.51986 (19)	0.62180 (7)	0.0252 (3)	
H27	0.4118	0.5787	0.5928	0.030*	
C28	0.40324 (5)	0.50458 (18)	0.69840 (7)	0.0242 (2)	
H28	0.4363	0.5508	0.7209	0.029*	
C29	0.36891 (5)	0.42094 (18)	0.74013 (7)	0.0242 (2)	
H29	0.3774	0.4059	0.7924	0.029*	
C30	0.32121 (5)	0.35891 (18)	0.70316 (7)	0.0238 (2)	
H30	0.2961	0.3023	0.7303	0.029*	
H1	0.9443 (7)	1.298 (2)	0.6418 (10)	0.040 (5)*	
H4	0.6953 (9)	1.112 (3)	0.4388 (13)	0.070 (7)*	
H8	0.6436 (8)	0.921 (3)	0.5477 (12)	0.064 (7)*	
H3	0.3919 (7)	0.693 (2)	0.3649 (10)	0.037 (5)*	
H5	0.2801 (7)	0.340 (2)	0.6060 (10)	0.040 (5)*	
H12	0.0205 (8)	−0.001 (3)	0.5722 (11)	0.052 (6)*	

### Atomic displacement parameters (Å^2^)

**Table:**

	*U*^11^	*U*^22^	*U*^33^	*U*^12^	*U*^13^	*U*^23^
Cl1	0.02091 (13)	0.03338 (16)	0.01460 (12)	−0.00592 (10)	0.00337 (9)	0.00278 (11)
Cl2	0.02201 (13)	0.02140 (13)	0.01473 (12)	−0.00058 (9)	0.00235 (9)	0.00273 (10)
Cl3	0.02047 (13)	0.03130 (15)	0.01329 (12)	−0.00316 (10)	0.00276 (9)	0.00268 (10)
Cl4	0.02048 (13)	0.02307 (13)	0.01446 (12)	−0.00125 (9)	0.00187 (9)	0.00367 (10)
Cl5	0.02339 (13)	0.02148 (13)	0.01490 (12)	−0.00022 (10)	0.00008 (9)	0.00332 (10)
Cl6	0.01993 (13)	0.03180 (15)	0.01370 (12)	−0.00260 (10)	0.00209 (9)	0.00197 (10)
O1	0.0137 (4)	0.0443 (5)	0.0221 (4)	−0.0085 (3)	0.0020 (3)	0.0002 (4)
O2	0.0153 (4)	0.0311 (4)	0.0170 (4)	−0.0058 (3)	−0.0026 (3)	0.0029 (3)
O3	0.0155 (4)	0.0264 (4)	0.0195 (4)	−0.0057 (3)	0.0034 (3)	−0.0006 (3)
O4	0.0137 (4)	0.0360 (5)	0.0185 (4)	−0.0030 (3)	−0.0026 (3)	0.0037 (4)
O5	0.0148 (4)	0.0416 (5)	0.0206 (4)	−0.0087 (3)	0.0009 (3)	0.0036 (4)
O6	0.0156 (4)	0.0296 (4)	0.0177 (4)	−0.0056 (3)	−0.0028 (3)	0.0023 (3)
O7	0.0163 (4)	0.0282 (4)	0.0195 (4)	−0.0075 (3)	0.0020 (3)	0.0000 (3)
O8	0.0135 (4)	0.0372 (5)	0.0169 (4)	−0.0064 (3)	−0.0024 (3)	0.0047 (4)
O9	0.0161 (4)	0.0313 (5)	0.0248 (4)	−0.0035 (3)	−0.0062 (3)	0.0067 (4)
O10	0.0159 (4)	0.0265 (4)	0.0179 (4)	−0.0047 (3)	0.0014 (3)	0.0001 (3)
O11	0.0167 (4)	0.0305 (4)	0.0198 (4)	−0.0041 (3)	−0.0036 (3)	0.0035 (3)
O12	0.0138 (4)	0.0323 (5)	0.0196 (4)	−0.0045 (3)	0.0003 (3)	0.0014 (3)
N1	0.0144 (4)	0.0246 (5)	0.0262 (5)	−0.0023 (4)	0.0010 (4)	−0.0009 (4)
N2	0.0249 (5)	0.0260 (5)	0.0233 (5)	−0.0044 (4)	0.0041 (4)	0.0014 (4)
N3	0.0153 (4)	0.0247 (5)	0.0225 (5)	−0.0032 (4)	−0.0015 (4)	−0.0013 (4)
N4	0.0262 (5)	0.0306 (6)	0.0216 (5)	−0.0059 (4)	−0.0017 (4)	0.0021 (4)
N5	0.0151 (4)	0.0238 (5)	0.0207 (5)	−0.0023 (4)	0.0001 (3)	−0.0016 (4)
N6	0.0214 (5)	0.0310 (5)	0.0189 (5)	−0.0043 (4)	0.0017 (4)	−0.0003 (4)
C1	0.0141 (5)	0.0205 (5)	0.0164 (5)	−0.0015 (4)	0.0018 (4)	−0.0019 (4)
C2	0.0143 (5)	0.0204 (5)	0.0120 (4)	−0.0022 (4)	0.0019 (3)	0.0002 (4)
C3	0.0134 (4)	0.0169 (5)	0.0152 (5)	−0.0013 (4)	0.0012 (4)	−0.0013 (4)
C4	0.0145 (4)	0.0143 (5)	0.0144 (5)	−0.0014 (4)	0.0016 (4)	−0.0022 (4)
C5	0.0159 (5)	0.0170 (5)	0.0133 (4)	0.0005 (4)	0.0009 (4)	0.0004 (4)
C6	0.0142 (5)	0.0188 (5)	0.0155 (5)	0.0014 (4)	−0.0004 (4)	−0.0012 (4)
C7	0.0138 (5)	0.0206 (5)	0.0152 (5)	−0.0011 (4)	0.0014 (4)	0.0002 (4)
C8	0.0152 (5)	0.0195 (5)	0.0123 (4)	−0.0006 (4)	0.0012 (4)	0.0002 (4)
C9	0.0146 (5)	0.0170 (5)	0.0145 (5)	−0.0009 (4)	0.0008 (4)	0.0000 (4)
C10	0.0144 (4)	0.0154 (5)	0.0153 (5)	−0.0014 (4)	0.0013 (4)	−0.0013 (4)
C11	0.0157 (5)	0.0170 (5)	0.0127 (4)	−0.0005 (4)	0.0013 (4)	0.0014 (4)
C12	0.0134 (5)	0.0193 (5)	0.0153 (5)	0.0004 (4)	−0.0006 (4)	−0.0002 (4)
C13	0.0157 (5)	0.0155 (5)	0.0172 (5)	0.0015 (4)	−0.0007 (4)	0.0010 (4)
C14	0.0162 (5)	0.0167 (5)	0.0133 (4)	0.0001 (4)	−0.0001 (4)	0.0014 (4)
C15	0.0156 (5)	0.0143 (5)	0.0158 (5)	−0.0006 (4)	0.0005 (4)	−0.0017 (4)
C16	0.0142 (5)	0.0158 (5)	0.0156 (5)	0.0001 (4)	0.0001 (4)	−0.0010 (4)
C17	0.0151 (5)	0.0186 (5)	0.0140 (5)	0.0001 (4)	0.0016 (4)	−0.0004 (4)
C18	0.0130 (4)	0.0170 (5)	0.0169 (5)	0.0006 (4)	0.0022 (4)	−0.0014 (4)
C19	0.0229 (6)	0.0258 (6)	0.0204 (5)	−0.0011 (4)	−0.0012 (4)	−0.0004 (5)
C20	0.0164 (5)	0.0262 (6)	0.0265 (6)	−0.0046 (4)	0.0017 (4)	−0.0065 (5)
C21	0.0228 (6)	0.0233 (6)	0.0241 (6)	−0.0060 (4)	0.0065 (4)	−0.0004 (5)
C22	0.0205 (5)	0.0246 (6)	0.0224 (6)	0.0013 (4)	−0.0006 (4)	0.0009 (5)
C23	0.0265 (6)	0.0303 (6)	0.0241 (6)	−0.0045 (5)	−0.0075 (5)	0.0015 (5)
C24	0.0164 (5)	0.0265 (6)	0.0309 (6)	−0.0037 (4)	−0.0019 (4)	−0.0038 (5)
C25	0.0213 (6)	0.0293 (6)	0.0252 (6)	−0.0060 (5)	0.0034 (4)	0.0002 (5)
C26	0.0209 (6)	0.0312 (6)	0.0205 (5)	−0.0034 (5)	−0.0034 (4)	0.0011 (5)
C27	0.0204 (6)	0.0325 (6)	0.0231 (6)	−0.0072 (5)	0.0038 (4)	−0.0011 (5)
C28	0.0176 (5)	0.0300 (6)	0.0238 (6)	−0.0038 (4)	−0.0016 (4)	−0.0052 (5)
C29	0.0240 (6)	0.0303 (6)	0.0172 (5)	−0.0025 (5)	−0.0015 (4)	−0.0008 (5)
C30	0.0212 (5)	0.0284 (6)	0.0217 (6)	−0.0041 (4)	0.0030 (4)	0.0003 (5)

### Geometric parameters (Å, °)

**Table:**

Cl1—C2	1.7293 (11)	C3—C4	1.5412 (15)
Cl2—C5	1.7218 (11)	C4—C5	1.4545 (14)
Cl3—C8	1.7262 (11)	C5—C6	1.3522 (15)
Cl4—C11	1.7192 (11)	C7—C8	1.4110 (14)
Cl5—C14	1.7305 (11)	C7—C12	1.5102 (15)
Cl6—C17	1.7168 (11)	C8—C9	1.3958 (15)
O1—C1	1.2408 (13)	C9—C10	1.5398 (15)
O2—C3	1.2517 (13)	C10—C11	1.4515 (14)
O3—C4	1.2193 (13)	C11—C12	1.3533 (15)
O4—C6	1.3218 (13)	C13—C14	1.4096 (15)
O4—H4	0.78 (2)	C13—C18	1.5069 (15)
O5—C7	1.2417 (13)	C14—C15	1.4040 (14)
O6—C9	1.2536 (13)	C15—C16	1.5408 (15)
O7—C10	1.2213 (13)	C16—C17	1.4481 (15)
O8—C12	1.3207 (12)	C17—C18	1.3520 (14)
O8—H8	0.80 (2)	C19—C20	1.3969 (17)
O9—C13	1.2476 (13)	C19—H19	0.9500
O10—C15	1.2491 (13)	C20—C21	1.3708 (17)
O11—C16	1.2218 (13)	C20—H20	0.9500
O12—C18	1.3232 (13)	C21—C22	1.3906 (16)
O12—H12	0.79 (2)	C21—H21	0.9500
N1—C22	1.3158 (16)	C22—H22	0.9500
N1—N2	1.3331 (15)	C23—C24	1.3994 (19)
N1—H1	0.888 (18)	C23—H23	0.9500
N2—C19	1.3189 (15)	C24—C25	1.3666 (18)
N3—C26	1.3144 (16)	C24—H24	0.9500
N3—N4	1.3355 (14)	C25—C26	1.3942 (16)
N3—H3	0.886 (17)	C25—H25	0.9500
N4—C23	1.3223 (16)	C26—H26	0.9500
N5—C30	1.3221 (16)	C27—C28	1.3966 (18)
N5—N6	1.3314 (14)	C27—H27	0.9500
N5—H5	0.891 (18)	C28—C29	1.3690 (17)
N6—C27	1.3236 (15)	C28—H28	0.9500
C1—C2	1.4069 (14)	C29—C30	1.3875 (16)
C1—C6	1.5079 (15)	C29—H29	0.9500
C2—C3	1.3913 (14)	C30—H30	0.9500
			
C6—O4—H4	113.0 (17)	C14—C13—C18	118.12 (9)
C12—O8—H8	112.9 (16)	C15—C14—C13	122.76 (10)
C18—O12—H12	110.5 (15)	C15—C14—Cl5	118.65 (8)
C22—N1—N2	125.20 (10)	C13—C14—Cl5	118.58 (8)
C22—N1—H1	122.0 (12)	O10—C15—C14	125.90 (10)
N2—N1—H1	112.8 (12)	O10—C15—C16	116.70 (9)
C19—N2—N1	115.76 (10)	C14—C15—C16	117.40 (9)
C26—N3—N4	125.10 (10)	O11—C16—C17	122.83 (10)
C26—N3—H3	121.5 (11)	O11—C16—C15	117.95 (10)
N4—N3—H3	113.4 (11)	C17—C16—C15	119.21 (9)
C23—N4—N3	115.44 (11)	C18—C17—C16	120.10 (10)
C30—N5—N6	124.80 (10)	C18—C17—Cl6	120.99 (8)
C30—N5—H5	120.8 (12)	C16—C17—Cl6	118.88 (8)
N6—N5—H5	114.4 (12)	O12—C18—C17	120.69 (10)
C27—N6—N5	115.74 (10)	O12—C18—C13	117.06 (9)
O1—C1—C2	125.21 (10)	C17—C18—C13	122.23 (10)
O1—C1—C6	116.67 (10)	N2—C19—C20	123.68 (11)
C2—C1—C6	118.09 (9)	N2—C19—H19	118.2
C3—C2—C1	122.90 (10)	C20—C19—H19	118.2
C3—C2—Cl1	119.09 (8)	C21—C20—C19	118.28 (11)
C1—C2—Cl1	118.00 (8)	C21—C20—H20	120.9
O2—C3—C2	124.89 (10)	C19—C20—H20	120.9
O2—C3—C4	116.93 (9)	C20—C21—C22	117.14 (11)
C2—C3—C4	118.17 (9)	C20—C21—H21	121.4
O3—C4—C5	123.41 (10)	C22—C21—H21	121.4
O3—C4—C3	118.33 (9)	N1—C22—C21	119.91 (11)
C5—C4—C3	118.24 (9)	N1—C22—H22	120.0
C6—C5—C4	120.45 (10)	C21—C22—H22	120.0
C6—C5—Cl2	120.77 (8)	N4—C23—C24	123.93 (12)
C4—C5—Cl2	118.72 (8)	N4—C23—H23	118.0
O4—C6—C5	121.29 (10)	C24—C23—H23	118.0
O4—C6—C1	116.75 (10)	C25—C24—C23	118.30 (11)
C5—C6—C1	121.96 (10)	C25—C24—H24	120.8
O5—C7—C8	125.43 (10)	C23—C24—H24	120.8
O5—C7—C12	116.43 (9)	C24—C25—C26	116.95 (12)
C8—C7—C12	118.14 (9)	C24—C25—H25	121.5
C9—C8—C7	122.55 (10)	C26—C25—H25	121.5
C9—C8—Cl3	119.72 (8)	N3—C26—C25	120.28 (11)
C7—C8—Cl3	117.69 (8)	N3—C26—H26	119.9
O6—C9—C8	125.73 (10)	C25—C26—H26	119.9
O6—C9—C10	116.17 (9)	N6—C27—C28	123.96 (12)
C8—C9—C10	118.09 (9)	N6—C27—H27	118.0
O7—C10—C11	122.72 (10)	C28—C27—H27	118.0
O7—C10—C9	118.35 (9)	C29—C28—C27	117.95 (11)
C11—C10—C9	118.92 (9)	C29—C28—H28	121.0
C12—C11—C10	119.98 (10)	C27—C28—H28	121.0
C12—C11—Cl4	121.19 (8)	C28—C29—C30	117.43 (11)
C10—C11—Cl4	118.79 (8)	C28—C29—H29	121.3
O8—C12—C11	121.01 (10)	C30—C29—H29	121.3
O8—C12—C7	116.74 (9)	N5—C30—C29	120.10 (11)
C11—C12—C7	122.25 (9)	N5—C30—H30	119.9
O9—C13—C14	125.75 (10)	C29—C30—H30	119.9
O9—C13—C18	116.14 (10)		
			
C22—N1—N2—C19	−0.02 (18)	C10—C11—C12—C7	−2.38 (17)
C26—N3—N4—C23	0.15 (19)	Cl4—C11—C12—C7	179.80 (8)
C30—N5—N6—C27	−0.32 (18)	O5—C7—C12—O8	0.75 (15)
O1—C1—C2—C3	−176.99 (12)	C8—C7—C12—O8	−179.96 (10)
C6—C1—C2—C3	1.03 (16)	O5—C7—C12—C11	−179.30 (11)
O1—C1—C2—Cl1	3.15 (17)	C8—C7—C12—C11	−0.01 (16)
C6—C1—C2—Cl1	−178.82 (8)	O9—C13—C14—C15	179.75 (11)
C1—C2—C3—O2	178.89 (11)	C18—C13—C14—C15	−0.34 (16)
Cl1—C2—C3—O2	−1.25 (16)	O9—C13—C14—Cl5	−1.12 (16)
C1—C2—C3—C4	−1.82 (16)	C18—C13—C14—Cl5	178.79 (8)
Cl1—C2—C3—C4	178.04 (8)	C13—C14—C15—O10	176.55 (11)
O2—C3—C4—O3	4.40 (15)	Cl5—C14—C15—O10	−2.58 (16)
C2—C3—C4—O3	−174.94 (10)	C13—C14—C15—C16	−3.15 (16)
O2—C3—C4—C5	−177.03 (10)	Cl5—C14—C15—C16	177.72 (7)
C2—C3—C4—C5	3.63 (14)	O10—C15—C16—O11	2.96 (15)
O3—C4—C5—C6	173.62 (11)	C14—C15—C16—O11	−177.31 (10)
C3—C4—C5—C6	−4.87 (15)	O10—C15—C16—C17	−175.99 (10)
O3—C4—C5—Cl2	−3.52 (15)	C14—C15—C16—C17	3.74 (15)
C3—C4—C5—Cl2	177.99 (8)	O11—C16—C17—C18	−179.53 (11)
C4—C5—C6—O4	−176.09 (10)	C15—C16—C17—C18	−0.63 (15)
Cl2—C5—C6—O4	1.00 (15)	O11—C16—C17—Cl6	−1.56 (15)
C4—C5—C6—C1	4.23 (16)	C15—C16—C17—Cl6	177.34 (8)
Cl2—C5—C6—C1	−178.68 (8)	C16—C17—C18—O12	178.68 (10)
O1—C1—C6—O4	−3.73 (15)	Cl6—C17—C18—O12	0.75 (15)
C2—C1—C6—O4	178.08 (10)	C16—C17—C18—C13	−3.07 (16)
O1—C1—C6—C5	175.96 (11)	Cl6—C17—C18—C13	179.00 (8)
C2—C1—C6—C5	−2.23 (16)	O9—C13—C18—O12	1.95 (15)
O5—C7—C8—C9	−179.02 (11)	C14—C13—C18—O12	−177.98 (10)
C12—C7—C8—C9	1.75 (16)	O9—C13—C18—C17	−176.36 (11)
O5—C7—C8—Cl3	−1.27 (16)	C14—C13—C18—C17	3.71 (16)
C12—C7—C8—Cl3	179.50 (8)	N1—N2—C19—C20	−0.85 (18)
C7—C8—C9—O6	178.05 (11)	N2—C19—C20—C21	0.50 (19)
Cl3—C8—C9—O6	0.34 (16)	C19—C20—C21—C22	0.68 (18)
C7—C8—C9—C10	−1.04 (16)	N2—N1—C22—C21	1.20 (19)
Cl3—C8—C9—C10	−178.74 (8)	C20—C21—C22—N1	−1.48 (18)
O6—C9—C10—O7	−1.84 (15)	N3—N4—C23—C24	−0.8 (2)
C8—C9—C10—O7	177.33 (10)	N4—C23—C24—C25	0.9 (2)
O6—C9—C10—C11	179.47 (10)	C23—C24—C25—C26	−0.24 (19)
C8—C9—C10—C11	−1.36 (15)	N4—N3—C26—C25	0.4 (2)
O7—C10—C11—C12	−175.60 (11)	C24—C25—C26—N3	−0.35 (19)
C9—C10—C11—C12	3.03 (15)	N5—N6—C27—C28	0.92 (19)
O7—C10—C11—Cl4	2.27 (15)	N6—C27—C28—C29	−0.4 (2)
C9—C10—C11—Cl4	−179.10 (8)	C27—C28—C29—C30	−0.63 (19)
C10—C11—C12—O8	177.57 (10)	N6—N5—C30—C29	−0.7 (2)
Cl4—C11—C12—O8	−0.24 (16)	C28—C29—C30—N5	1.20 (19)

### Hydrogen-bond geometry (Å, °)

**Table:**

*D*—H···*A*	*D*—H	H···*A*	*D*···*A*	*D*—H···*A*
N1—H1···O2	0.889 (17)	1.753 (17)	2.6138 (13)	162.2 (17)
N1—H1···O3	0.889 (17)	2.578 (18)	3.1583 (14)	123.7 (14)
N3—H3···O6	0.886 (18)	1.811 (17)	2.6621 (13)	160.4 (17)
N3—H3···O7	0.886 (18)	2.431 (18)	3.0066 (14)	123.0 (14)
O4—H4···O1	0.79 (2)	2.26 (2)	2.6619 (13)	113 (2)
O4—H4···O5	0.79 (2)	1.94 (2)	2.6723 (12)	155 (2)
N5—H5···O10	0.890 (18)	1.881 (18)	2.7450 (14)	163.2 (16)
N5—H5···O11	0.890 (18)	2.410 (17)	2.9659 (13)	120.7 (14)
O8—H8···O1	0.80 (2)	1.86 (2)	2.5930 (12)	152 (2)
O8—H8···O5	0.80 (2)	2.25 (2)	2.6582 (13)	112.7 (18)
O12—H12···O9	0.79 (2)	2.23 (2)	2.6595 (13)	115.1 (18)
O12—H12···O9^i^	0.79 (2)	2.02 (2)	2.6801 (12)	142 (2)
C20—H20···O2^ii^	0.95	2.57	3.1097 (15)	116
C21—H21···O3^iii^	0.95	2.36	3.2754 (15)	162
C22—H22···O3	0.95	2.60	3.1913 (15)	121
C25—H25···O10	0.95	2.47	3.4056 (15)	169
C26—H26···O7	0.95	2.44	3.0363 (15)	121
C26—H26···N6	0.95	2.51	3.2665 (17)	137
C27—H27···O7	0.95	2.37	3.2765 (15)	159
C28—H28···O6^iv^	0.95	2.47	3.1091 (15)	124
C30—H30···O11	0.95	2.34	2.9551 (15)	122

Symmetry codes: (i) −*x*, −*y*, −*z*+1; (ii) −*x*+2, *y*+1/2, −*z*+3/2; (iii) −*x*+2, −*y*+3, −*z*+1; (iv) *x*, −*y*+3/2, *z*+1/2.
